# Effect of a diet rich in galactose or fructose, with or without fructooligosaccharides, on gut microbiota composition in rats

**DOI:** 10.3389/fnut.2022.922336

**Published:** 2022-08-12

**Authors:** Nor Adila Mhd Omar, Johan Dicksved, Johanita Kruger, Galia Zamaratskaia, Karl Michaëlsson, Alicja Wolk, Jan Frank, Rikard Landberg

**Affiliations:** ^1^Unit of Cardiovascular and Nutritional Epidemiology, Institute of Environmental Medicine, Karolinska Institutet, Stockholm, Sweden; ^2^Department of Animal Nutrition and Management, Swedish University of Agricultural Sciences, Uppsala, Sweden; ^3^Department of Food Biofunctionality, Institute of Nutritional Sciences, University of Hohenheim, Stuttgart, Germany; ^4^Department of Molecular Sciences, Swedish University of Agricultural Sciences, Uppsala, Sweden; ^5^Department of Surgical Sciences, Uppsala University, Uppsala, Sweden; ^6^Department of Public Health and Clinical Medicine, Nutritional Research. Umeå University, Umeå, Sweden; ^7^Division of Food and Nutrition Science, Department of Biology and Biological Engineering, Chalmers University of Technology, Gothenburg, Sweden

**Keywords:** fructose, galactose, fructooligosaccharides, gut microbiota, 16S rRNA

## Abstract

Recent studies suggest that a diet rich in sugars significantly affects the gut microbiota. Adverse metabolic effects of sugars may partly be mediated by alterations of gut microbiota and gut health parameters, but experimental evidence is lacking. Therefore, we investigated the effects of high intake of fructose or galactose, with/without fructooligosaccharides (FOS), on gut microbiota composition in rats and explored the association between gut microbiota and low-grade systemic inflammation. Sprague–Dawley rats (*n* = 6/group) were fed the following isocaloric diets for 12 weeks (% of the dry weight of the sugars or FOS): (1) starch (control), (2) fructose (50%), (3) galactose (50%), (4) starch+FOS (15%) (FOS control), (5) fructose (50%)+FOS (15%), (6) galactose (50%)+FOS (15%), and (7) starch+olive (negative control). Microbiota composition in the large intestinal content was determined by sequencing amplicons from the 16S rRNA gene; 341F and 805R primers were used to generate amplicons from the V3 and V4 regions. Actinobacteria, Verrucomicrobia, Tenericutes, and Cyanobacteria composition differed between diets. *Bifidobacterium* was significantly higher in all diet groups where FOS was included. Modest associations between gut microbiota and metabolic factors as well as with gut permeability markers were observed, but no associations between gut microbiota and inflammation markers were observed. We found no coherent effect of galactose or fructose on gut microbiota composition. Added FOS increased Bifidobacterium but did not mitigate potential adverse metabolic effects induced by the sugars. However, gut microbiota composition was associated with several metabolic factors and gut permeability markers which warrant further investigations.

## Introduction

The gut microbiota in the large intestine of humans and animals comprises more than one trillion microorganisms but is dominated by two major bacterial phyla, Firmicutes and Bacteriodetes (1, 2). Studies have demonstrated that metabolic activity of the gut microbiota is essential in maintaining host homeostasis and health (3, 4). In recent years, probiotic bacteria have received escalating attention, particularly for their beneficial health effects on the host through various mechanisms including modulation of gut microbiota, metabolic effects, and regulation of immune responses (5, 6). Among probiotics, *Bifidobacterium* and *Lactobacillus* genera are of major importance due to their functional properties (5). Probiotics have also been studied for anticarcinogenic (7), antipathogenic (8), and anticholesterolemic (9) activities.

Prebiotics represent important factors affecting gut microbiota composition, richness, and activity, and their role in human health (10, 11). Prebiotics are carbohydrates such as dietary fibers including fructooligosaccharides, galactooligosaccharides, and inulin (12). Prebiotics are resistant to digestion in the upper sections of the alimentary tract and undergo fermentation by saccharolytic bacteria such as *Bifidobacterium* in the intestine (13). Therefore, the change in diet by including prebiotics can result in a significant change in the microbiota after only 24 h (10, 14), although short-term dietary changes are typically transient, long-term dietary changes alter the microbiota composition more robustly (10, 14). Interestingly, studies have suggested alterations in gut microbiota associated with the consumption of sugar and sugar-sweetened beverages in humans, but firm evidence is lacking (15).

Studies in rats have shown that the gut microbiota was significantly affected within a week after feeding simple sugars including a decrease in diversity (16). Common sugars in the diet, such as glucose, fructose, and galactose, are actively absorbed in the small intestine, but the degree of absorption varies with the type of sugar and dose (17, 18). For example, excessive intake of fructose may lead to incompletely digested fructose reaching the colon, where intestinal discomfort and even negative health outcomes due to inappropriate immune response, may result consequently (19, 20). Studies have suggested that high consumption of fructose in the diet could affect the microbial community and lead to an increase in pathogenic bacteria, deterioration of the intestinal barrier function, reduced mucus thickness, and a subsequent increase in translocation of microbiota and increased concentrations of endotoxin in the bloodstream (21, 22). Moreover, consumption of fructose, either in solid or liquid form, has been shown to affect microbiota composition differently (23).

In rodents, chronic intake of galactose is known to cause toxicity due to the accumulation of reactive oxygen species and advanced glycation end products (AGE) (24). Feeding D-galactose to accelerate aging is a well-established animal model (25). In addition, a diet containing 15% galactose has been found to decrease the abundance of Firmicutes, alter the Firmicutes:Bacteroidetes ratio, and decrease the abundance of *Clostridium coccoides* in rats, compared with diets with 15% glucose or 15% fructose (26). D-galactose-induced aging has been shown to modulate gut microbiota composition significantly at the phylum level in rats (27–29).

A high-fiber diet may provide beneficial health effects through stimulation of the growth of specific gut microbiota (30–33). Prebiotic fiber types, such as fermentable fructooligosaccharides (FOS) and inulin, have consistently been shown to increase the abundance of *Bifidobacterium* and lactic acid bacteria (LAB) in the large intestine. They have also been shown to suppress the growth of pathogenic bacteria that may result in endotoxemia, and could thereby provide a positive effect on gut barrier integrity and lower sub-clinical systemic inflammatory responses (34, 35). A high intake of fermentable dietary fiber increases the production of short-chain fatty acids (SCFA), whereas reduced production of SCFA has been reported at a low intake of fermentable dietary fiber (36). SCFA, that is, acetate, propionate, and butyrate, modulate the secretion of hormones, insulin sensitivity, and immune responses (37, 38). Low intake of dietary fiber and high intake of sugar are believed to have detrimental effects on microbial diversity and human health (39, 40).

The main hypothesis tested in this study was that a high intake of simple sugars, such as fructose or galactose, causes malabsorption in the small intestine so that excess sugars reach the large intestine, causing unfavorable alterations in gut microbiota composition and activity (12, 41, 42). A second hypothesis tested was that fermentable dietary fiber, such as FOS, counteracts these adverse effects of simple sugars through modulation of the gut microbiota (43). We, therefore, investigated the impact of diets high in fructose or galactose on gut microbiota composition and examined whether simultaneous administration of FOS could mitigate the potential adverse effects of the simple sugars over a 12-week intervention. We also assessed associations between gut microbiota diversity, metabolic factors, and inflammation and gut permeability biomarkers.

## Methods

### Animals and diets

Samples and data from a previous 12-week rat study, where we investigated the metabolic effects of high sugar diets with and without the addition of FOS, were used for the investigations in the present study (44). The estimated number of animals per group was six, to differentiate an assumed effect of 30% difference (100 *vs*. 140 units) between treatments and control. A standard deviation of 15 units in 12 comparisons provides a *p* < 0.001 and power of 0.9. In brief, healthy male Sprague–Dawley rats (*n* = 90) were purchased from Janvier Labs at age 7 weeks with an initial body weight of 250–274 g and were randomly assigned to one of seven diet groups (*n* = 12 per group) as follows: (1) fructose, (2) fructose+FOS, (3) galactose, (4) galactose+FOS, (5) starch (control), (6) starch+FOS, and (7) starch+olive (negative control), and one baseline group (*n* = 6) sacrificed before the commencement of the feeding trial. Six out of 12 rats in each group were sacrificed at the end of week 6 and the other six at the end of week 12. The animal experiment (trial no. V 351/18 BC) was approved by the Regional Council Stuttgart (Baden-Württemberg, Germany) ethics committee. All animal procedures were carried out in accordance with the Federation of European Laboratory Animal Science Association (FELASA) guidelines for the care and use of laboratory animals. The animal studies are reported in accordance and compliance with the ARRIVE guidelines (45).

The experimental diets contained different carbohydrates in isocaloric conditions. The high-carbohydrate diets consisted of 50% fructose or 50% galactose, while the diets with added FOS (inulin-type, DP4-5, MW: 624–679 from chicory root with 95% purity, Boneo GmbH, Germany) contained 15% FOS (all by weight). Starch (native starch, The Carl Roth GmbH+Co.KG, Germany), was used instead of sugars for the control diet and was added to all other diets in varying quantities to adjust total energy intake in order to obtain isocaloric conditions. All diets were prepared with 6% safflower oil as a source of n-6 polyunsaturated fatty acids (PUFA) except for the negative control diet, which used 6% olive oil (low in n-6). Starch+FOS was used as the FOS control diet. The dietary regimen for the groups fed galactose had to be modified due to adverse health effects observed in these rats after 2 weeks of intervention. For the remainder of the study, the rats in the galactose and galactose+FOS groups were given the respective diets for 4 days, followed by 3 days on the starch diet (control). All rats had access to diets and water *ad libitum*. Body weight and feed intake were recorded weekly. At the end of the respective feeding period, the rats were fasted for 12 h, anesthetized with carbon dioxide gas, and killed by decapitation. Intestinal contents were collected in Eppendorf tubes and immediately stored at −80°C. Blood samples were collected into heparinized monovettes (Monovette, Sarstedt, Germany) at 6 and 12 weeks, and glucose concentrations were measured immediately. Plasma and serum were separated from the blood cells immediately and stored at −80°C after collection. Metabolic factors, inflammation and gut permeability biomarkers, and AGEs were analyzed in plasma samples and the results are reported in our previous study (44), from which data were available for the present study and linked to effects on the gut microbiota.

### Gut microbiota analysis

DNA was extracted in singlet samples from the intestinal sample contents of all animals included in the study using QIAamp Fast DNA Stool mini kit (Qiagen, Hilden, Germany) according to the manufacturer's protocol, with the exception that the bacterial cell walls were mechanically disrupted with 0.1 mm zirconium/silica beads (Biospec Products) for 2 x 60 s using a Precellys Evolution device (Bertin Technologies, Montigny-le-Bretonneaux, France).

Amplicons from the V3 and V4 regions of the 16S rRNA gene were generated from the extracted DNA using the primers 341F and 805R. For the PCR reactions, Phusion High-Fidelity PCR Master Mix (New England Biolabs, United States) was used and the PCR products were purified with Qiagen Gel Extraction Kit (Qiagen) and quantified with Qubit 3.0 Fluorometer (Invitrogen, Thermo Fisher Scientific). The final libraries were generated with a NEBNext Ultra DNA Library Prep Kit that incorporated barcodes and adaptors. The amplicons were then sequenced on the Illumina platform at Novogene (Beijing, China).

### Bioinformatic analysis

Generated paired-end reads were first assigned to samples based on their unique barcode sequence. Reads were then merged after truncating the barcode and primer sequence using FLASH (v1.2.7, http://ccb.jhu.edu/software/FLASH/) (46). Merged sequences were analyzed using QIIME (v1.7.0) (47, 48). Sequence analysis by clustering of operational taxonomic units (OTUs) was performed using Uparse software (Uparse) (49), with sequences with ≥97% homology assigned to the same OTU. The representative sequences in each OTU were then annotated using the SILVA Database (http://www.arb-silva.de/) for species annotation at each taxonomic rank (50, 51).

### Statistical analysis

The statistical analyses were carried out using SAS statistical analysis software (release 9.4; SAS Institute, Cary, NC, United States). The effect of diet on microbiota composition at phylum and genus level was evaluated using one-way analysis of variance (ANOVA) followed by Tukey's multiple comparisons, with differences at 12 weeks defined as the primary outcome. A secondary analysis was carried out to evaluate the effects on 6 weeks if effects of specific diets were found after 12 weeks of intervention. Relative abundance at genus level >5% was used as the cut-off for inclusion in statistical testing. Assumption of normality and homogeneity of variance were assessed using the Shapiro–Wilks test. Data not normally distributed were log-transformed before analysis. All data analyzedusing the generalized linear model are presented as least square (LS) means ± standard error of the mean (SEM), adjusted for baseline values. Values of *p* < 0.05 were considered statistically significant. Correlations between the most abundant microbial phyla and metabolic factors and inflammation-related markers were assessed by Spearman's rank correlation tests.

## Results

### Characteristics of energy intake and initial and final body weight of the rats

Data were obtained for 90 rats treated with a high-fructose or high-galactose diet, with or without added FOS, or fed a control diet (starch, starch+FOS, or olive oil control) for 12 weeks (*n* = 6 rats/group). Energy intake was significantly higher in rats fed fructose than in rats fed starch+FOS. The rats in all intervention groups had an initial body weight of 251–258 g. Body weight after the 12-week intervention was significantly lower in the galactose and galactose+FOS groups compared with the starch (control), starch+olive (negative control), and fructose groups (Table 1).

**Table 1:** Descriptive characteristics of the rats fed a control diet (starch, starch + FOS, starch+olive) or a high-fructose or high-galactose diet, with and without added fructooligosaccharides (FOS), after 12 weeks of intervention.

	**Starch (control)**	**Starch + olive** **(negative control)**	**Starch + FOS** **(FOS control)**	**Fructose**	**Fructose + FOS**	**Galactose**	**Galactose + FOS**
Initial body weight (g)	258.3 ± 1.8	254.0 ± 2.3	252.9 ± 2.4	254.7 ± 2.4	253.2 ± 3.3	256.7 ± 2.5	251.7 ± 2.9
Final body weight (g)	605.8 ± 6.7^a^	587.5 ± 21.5^a^	559.0 ± 20.1^b^	598.8 ± 13.4^a^	553.2 ± 17.6^b^	498.0 ± 15.5^c^	479.8 ± 16.6^c^
Energy intake (kcal/day)	380.3 ± 8.2^ab^	369.5 ± 9.4^abc^	318.6 ± 14.7^c^	426.2 ± 9.9^a^	350.2 ± 8.9^bc^	393.8 ± 19.9^ab^	403.6 ± 20.8^ab^
FOS intake (g/day)	n.a	n.a	3.5 ± 0.1	n.a	3.9 ± 0.1	n.a	4.5 ± 0.2
Fructose intake (g/day)	n.a	n.a	n.a	15.2 ± 0.4	12.9 ± 0.3	n.a	n.a
Galactose intake (g/day)	n.a	n.a	n.a	n.a	n.a	14.0 ± 0.7	14.9 ± 0.8

*n.a, not applicable. Values shown are LS mean ± SEM of six rats. Groups were assessed by one-way ANOVA followed by Tukey's test. Means with different superscripts (lowercase letters) differ significantly (p <0.05)*.

### Effects of diet on alpha-diversity of the gut microbiota

**Figure 1:**
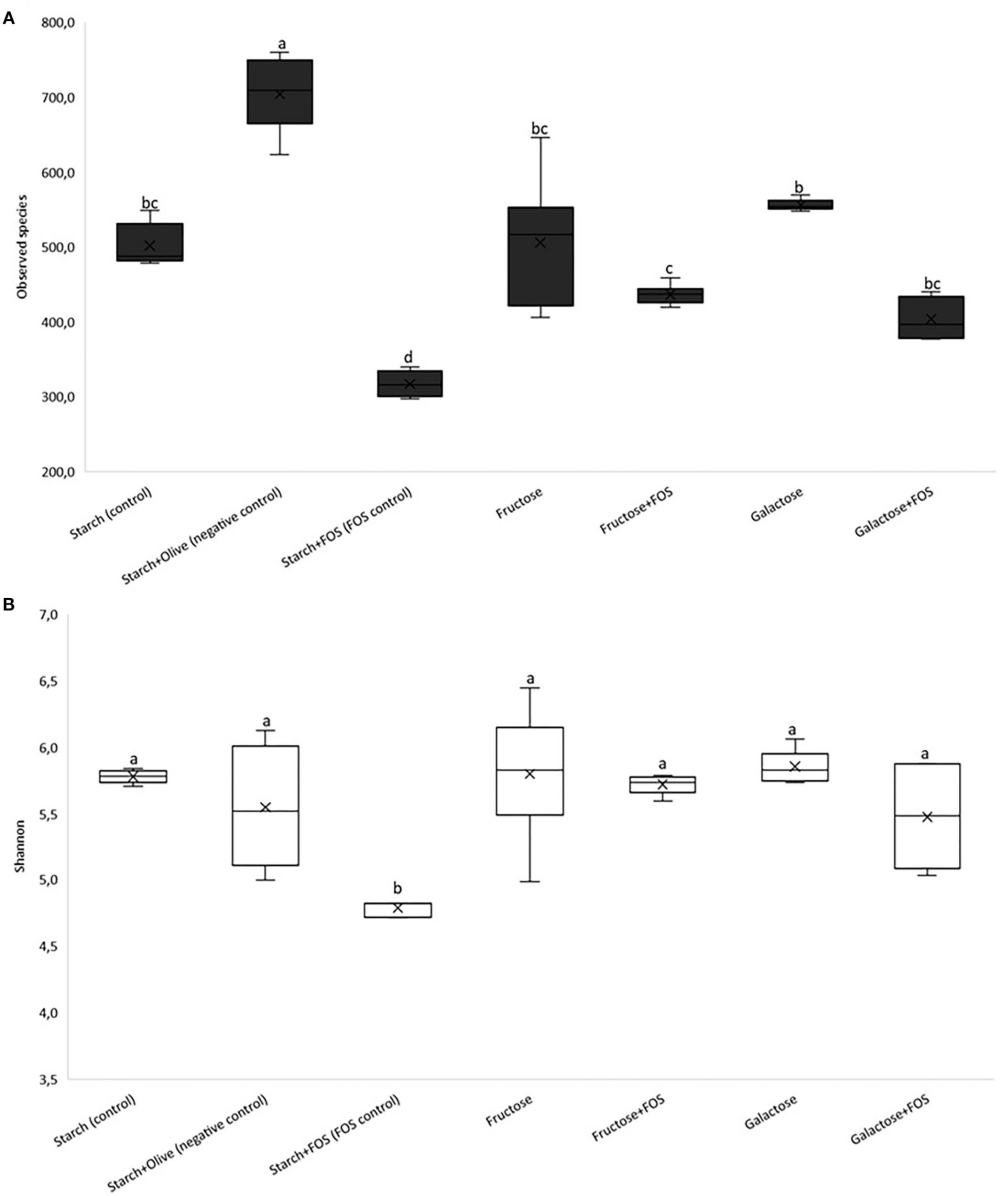
Results of 16S rRNA gene sequence analysis of the gut microbiota community in the large intestine of rats fed a control diet (starch, starch+FOS, starch+olive) or a high-fructose or high-galactose diet, with and without added fructooligosaccharides (FOS), after 12 weeks. Alpha diversity; **(A)** observed species, **(B)** Shannon's diversity index. Values shown are LS mean ± SEM of six rats. Groups were assessed by one-way ANOVA followed by Tukey's test. Means with different superscripts (lowercase letters) differ significantly (*p* < 0.05).

The baseline richness of bacterial OTUs and Shannon diversity is shown in Figures 1A,B, respectively. Significantly lower diversity was observed in the starch+FOS group compared with all other diet groups after 12 weeks of intervention (Figure 1A). Similarly, Shannon index was significantly lower in the starch+FOS compared with the other diet groups (Figure 1B). At 6 weeks, no similar difference in alpha-diversity was observed (data not shown).

### Effects of diet on gut microbiota composition at the phylum and genus levels

**Figure 2:**
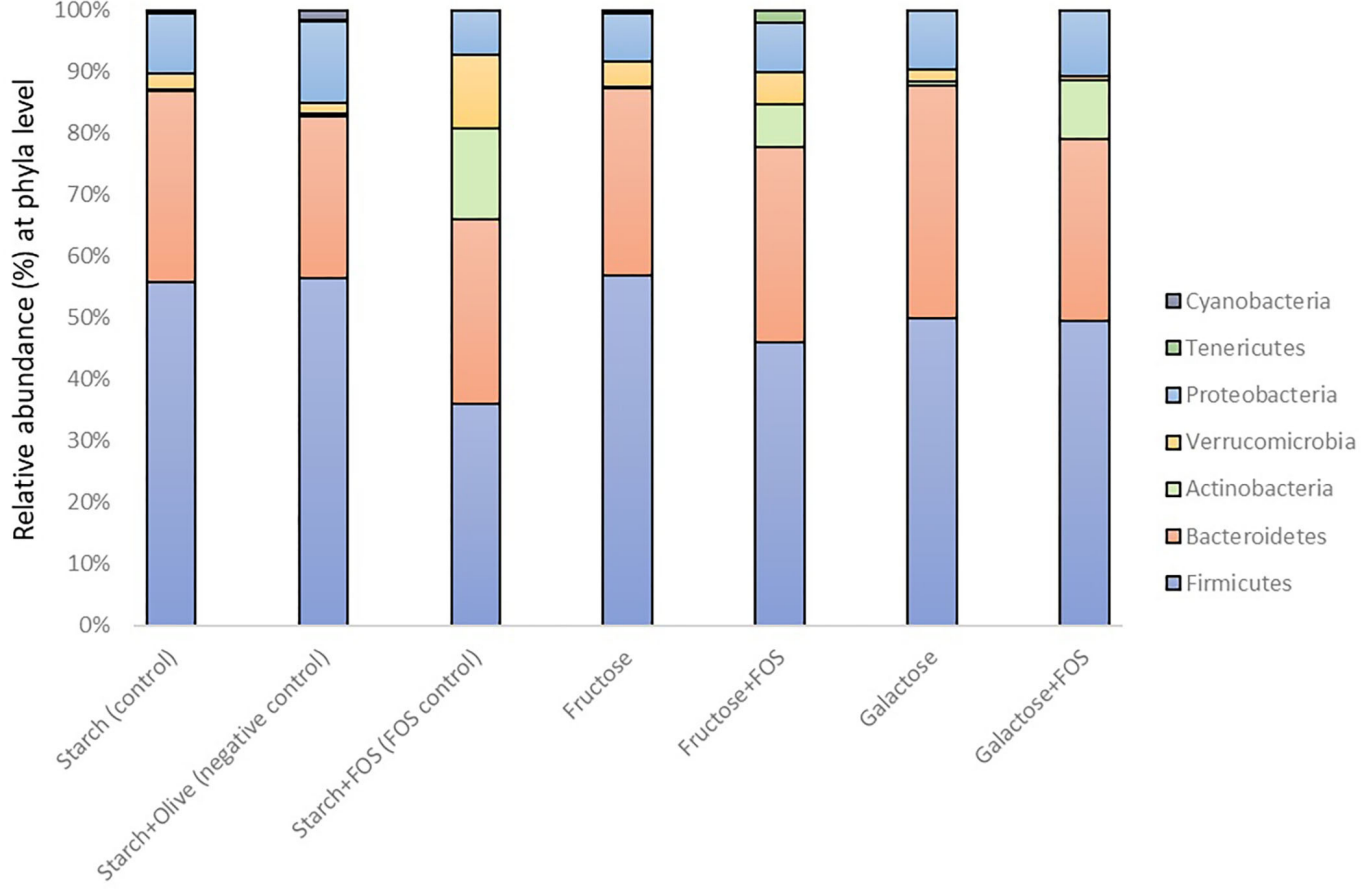
Gut microbiota composition (relative abundance, %) at phylum level in rats fed a control diet (starch, starch+FOS, starch+olive) or a high-fructose or high-galactose diet, with and without added fructooligosaccharides (FOS), after 12 weeks.

**Figure 3:**
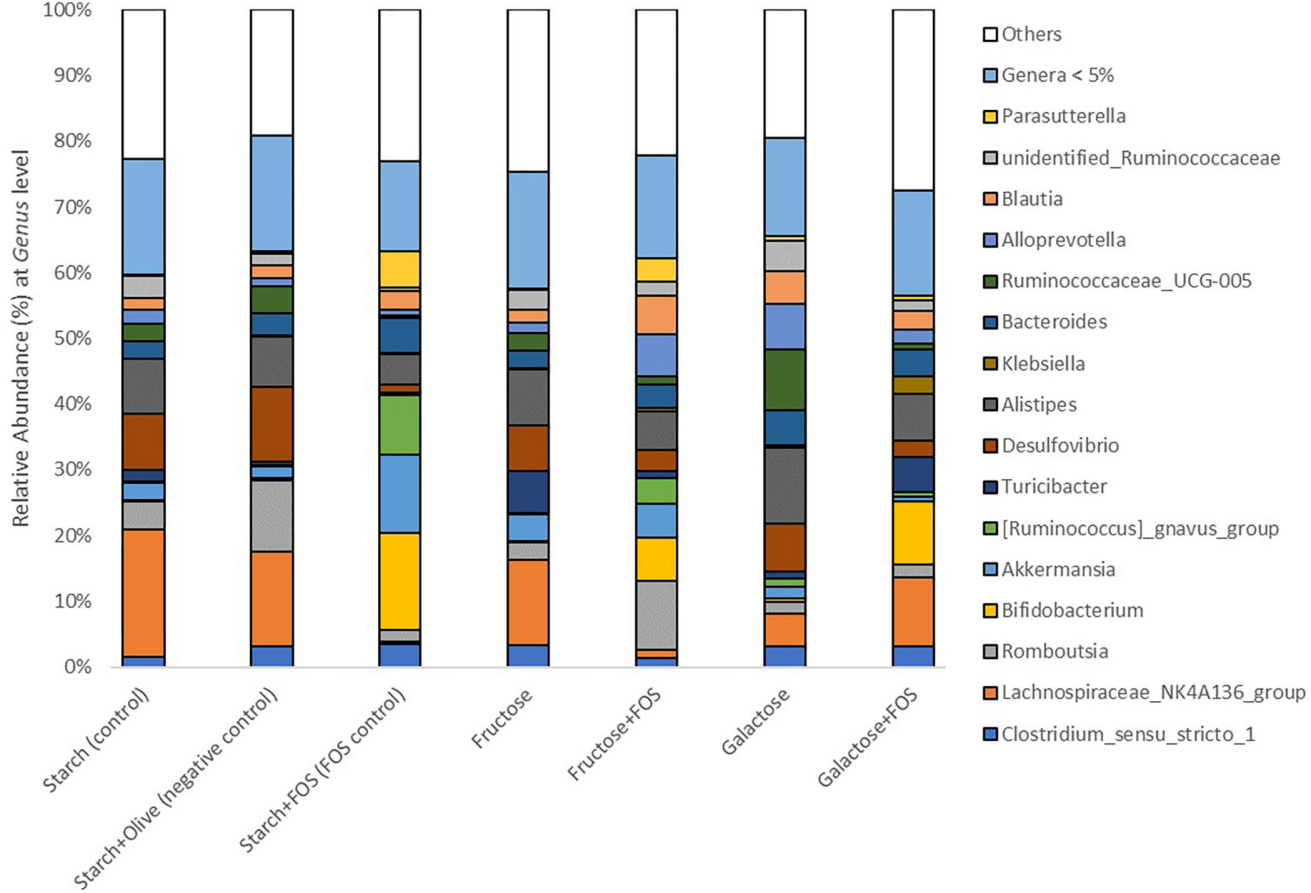
Gut microbiota composition (relative abundance, %) at phylum level in rats fed a control diet (starch, starch+FOS, starch+olive) or a high-fructose or high-galactose diet, with and without added fructooligosaccharides (FOS), after 12 weeks. Only classified genera with relative abundance above 5.0% cut-off level are shown. White bars indicate all genera with mean relative abundance <5.0%.

Taxonomically, over 300 genera belonging to 22 phyla were identified in the large intestine of the rats, of which 16 genera with relative abundance >5.0% comprised >50% of the variation in microbiota (Figures 2, 3). The microbiota in the large intestine of the rats at baseline was dominated by the phyla Firmicutes (55%), Bacteroidetes (37%), and Proteobacteria (5%) (Figure 2, Table 2).

**Table 2:** Effects of high-carbohydrate diets (fructose and galactose), with and without additional fructooligosaccharides (FOS), on relative abundance (%) of major phyla in the large intestine microbiota of rats after 12 weeks.

	**Diets**							
	**Baseline**	**Starch** **(Control)**	**Starch + olive ** **(negative control)**	**Starch + FOS** **(FOS control)**	**Fructose**	**Fructose + FOS**	**Galactose**	**Galactose + FOS**
Firmicutes	54.58 ± 1.44	55.72 ± 1.86	56.54 ± 6.36	36.13 ± 8.66	56.87 ± 2.71	46.03 ± 3.92	50.02 ± 2.46	49.44 ± 6.06
Bacteroidetes	37.08 ± 1.66	31.18 ± 2.25	26.14 ± 5.82	29.80 ± 10.30	30.45 ± 2.94	31.71 ± 3.57	37.78 ± 2.75	29.60 ± 2.73
Actinobacteria	1.25 ± 0.46	0.16 ± 0.03^d^	0.59 ± 0.18^cd^	14.87 ± 1.86^a^	0.25 ± 0.07^d^	6.91 ± 1.16^bc^	0.69 ± 0.14^cd^	9.6 ± 3.25^ab^
Verrucomicrobia	0.39 ± 0.15	2.74 ± 1.29^b^	1.74 ± 0.50^b^	11.94 ± 4.13^a^	4.13 ± 1.7^b^	5.23 ± 1.27^ab^	1.89 ± 1.17^b^	0.68 ± 0.15^b^
Proteobacteria	5.32 ± 0.69	9.69 ± 1.23	13.07 ± 3.28	7.20 ± 1.73	7.82 ± 0.95	8.12 ± 1.63	9.48 ± 1.68	10.63 ± 4.3
Tenericutes	0.09 ± 0.01	0.03 ± 0.01^b^	0.30 ± 0.07^ab^	0.03 ± 0.01^ab^	0.04 ± 0.02^b^	1.96 ± 0.07^a^	0.05 ± 0.01^b^	0.02 ± 0.01^b^
Cyanobacteria	1.28 ± 0.54	0.48 ± 0.15^b^	1.60 ± 0.51^a^	0.01 ± 0.00^b^	0.44 ± 0.25^b^	0.03 ± 0.01^b^	0.08 ± 0.03^b^	0.02 ± 0.00^b^

*Values shown are LS mean ± SEM of six rats (percentage of relative abundance). Groups were assessed by one-way ANOVA followed by Tukey's test. Means with different superscripts (lowercase letters) differ significantly (p <0.05)*.

In general, a significantly higher relative abundance of Actinobacteria was observed in the starch+FOS and galactose+FOS groups compared with the fructose, galactose, starch (control), and starch+olive (negative control) groups. Tenericutes abundance was significantly higher in the fructose+FOS group than in the starch (control), fructose, galactose, and galactose+FOS groups at 12 weeks of intervention. However, no similar difference in the abundance of Actinobacteria and Tenericutes was observed at 6 weeks (data not shown).

The relative abundance of Verrucomicrobia was significantly higher in the starch+FOS group than in all other diet groups except the fructose+FOS group. A similar difference was observed in the relative abundance of Verrucomicrobia (*p* < 0.05) at 6 weeks (data not shown).

Cyanobacteria abundance was significantly higher in the starch+olive (negative control) group than in the other diet groups after 12 weeks of intervention (Table 2), but no similar difference was observed at 6 weeks (data not shown). No differences in the effect of diets on microbiota composition were observed for Firmicutes, Bacteroidetes, and Proteobacteria after 12 weeks of intervention.

At the genus level, the relative abundance of *Lachnospiraceae_NK4A136*_group*, Bifidobacterium, Akkermansia, [Ruminococcus]_gnavus*_group *Desulfovibrio, Klebsiella, Ruminococcaceae_UCG-005, Alloprevotella*, unidentified*_Ruminococcaceae*, and *Parasutterella* differed significantly between the diets at 12 weeks of intervention (Table 3, Figure 3). The relative abundance of *Lachnospiraceae_NK4A136*_group was significantly higher in rats fed starch (control), starch+olive (negative control), fructose, and galactose+FOS diets compared with the other diet groups (Table 3, Figure 3). The relative abundance of *Bifidobacterium, Akkermansia*, and *[Ruminococcus]_gnavus*_group were significantly higher in rats fed the starch+FOS diet compared to the other diet groups, except for galactose+FOS, where the abundance of *Bifidobacterium* did not differ significantly from the starch+FOS diet group at 12 weeks (Table 3). However, the relative abundance of *Bifidobacterium* was significantly higher in the fructose+FOS and galactose groups than in the other diet groups. However, no similar difference was observed in these genera at 6 weeks of intervention (data not shown).

**Table 3:** Effects of high-carbohydrate diets (fructose and galactose), with and without additional fructooligosaccharides (FOS), on relative abundance (%) of the genus in the large intestine microbiota of rats after 12 weeks.

	**Baseline**	**Diets**						
		**Starch (Control)**	**Starch+olive (negative control)**	**Starch+FOS (FOS control)**	**Fructose**	**Fructose+FOS**	**Galactose**	**Galactose+FOS**
*Clostridium_ sensu_stricto _1*	3.60 ± 0.55	1.48 ± 0.49	3.1 ± 1.59	3.49 ± 2.03	3.33 ± 1.31	1.30 ± 0.26	3.05 ± 0.51	3.05 ± 1.09
*Lachnospiraceae_NK4A136_group*	7.29 ± 0.91	19.48 ± 2.79^d^	14.37 ± 3.81^cd^	0.29 ± 0.11^a^	12.97 ± 2.82^bcd^	1.22 ± 0.31^ab^	5.04 ± 0.89^abc^	10.51 ± 3.74^abcd^
*Romboutsia*	8.26 ± 1.31	4.29 ± 1.06	10.87 ± 4.77	1.85 ± 0.38	2.71 ± 0.65	10.52 ± 2.09	1.74 ± 0.24	2.09 ± 0.27
*Bifidobacterium*	1.04 ± 0.45	0.09 ± 0.02^a^	0.43 ± 0.17^ab^	14.69 ± 1.84^d^	0.16 ± 0.08^ab^	6.61 ± 1.11^bc^	0.56 ± 0.12^ab^	9.52 ± 3.25^cd^
*Akkermansia*	0.39 ± 0.15	2.74 ± 1.29^a^	1.74 ± 0.50^a^	11.94 ± 4.13^b^	4.13 ± 1.70^a^	5.23 ± 1.27^ab^	1.89 ± 1.17^a^	0.68 ± 0.15^a^
*[Ruminococcus]_gnavus_group*	0.09 ± 0.01	0.11 ± 0.01^a^	0.20 ± 0.05^a^	9.12 ± 6.01^b^	0.13 ± 0.02^a^	3.85 ± 0.64^ab^	1.09 ± 0.27^a^	0.69 ± 0.18^a^
*Turicibacter*	2.90 ± 0.92	1.83 ± 0.65	0.59 ± 0.20	0.36 ± 0.07	6.35 ± 3.99	1.04 ± 0.33	1.18 ± 0.31	5.41 ± 1.64
*Desulfovibrio*	2.89 ± 0.65	8.49 ± 1.12^ab^	11.27 ± 3.40^b^	1.31 ± 0.56^a^	6.97 ± 1.12^ab^	3.16 ± 1.56^a^	7.28 ± 1.74^ab^	2.42 ± 0.54^a^
*Alistipes*	10.29 ± 1.06	8.30 ± 0.58	7.73 ± 2.05	4.54 ± 2.25	8.50 ± 0.82	5.94 ± 1.36	11.51 ± 1.76	7.09 ± 1.91
*Klebsiella*	0.06 ± 0.02	0.07 ± 0.03^a^	0.17 ± 0.09^a^	0.14 ± 0.05^a^	0.21 ± 0.09^a^	0.59 ± 0.13^ab^	0.31 ± 0.14^a^	2.79 ± 1.37^b^
*Bacteroides*	5.90 ± 1.08	2.60 ± 0.37	3.42 ± 0.80	5.41 ± 1.59	2.63 ± 0.47	3.57 ± 0.68	5.39 ± 0.59	4.03 ± 0.69
*Ruminococcaceae_UCG-005*	3.03 ± 0.57	2.75 ± 0.58^a^	4.06 ± 1.06^a^	0.37 ± 0.02^a^	2.78 ± 0.45^a^	1.23 ± 1.14^a^	9.30 ± 2.17^b^	0.93 ± 0.24^a^
*Alloprevotella*	4.17 ± 0.61	2.19 ± 0.28^a^	1.30 ± 0.22^a^	0.82 ± 0.11^a^	1.50 ± 0.33^a^	6.40 ± 1.13^b^	6.83 ± 1.36^b^	2.11 ± 0.35^a^
*Blautia*	2.84 ± 0.86	1.72 ± 0.22	1.90 ± 0.59	2.88 ± 0.94	2.07 ± 0.32	5.82 ± 1.92	5.01 ± 0.59	2.78 ± 1.12
Unidentified_ *Ruminococcaceae*	1.99 ± 0.37	3.39 ± 0.16^bc^	1.79 ± 0.40^ab^	0.53 ± 0.08^a^	2.90 ± 0.61^bc^	2.14 ± 0.64^ab^	4.67 ± 0.40^c^	1.61 ± 0.29^ab^
*Parasutterella*	0.77 ± 0.14	0.19 ± 0.03^a^	0.26 ± 0.04^a^	5.49 ± 1.21^c^	0.17 ± 0.05^a^	3.58 ± 0.24^b^	0.72 ± 0.17^a^	0.81 ± 0.27^a^

*Values shown are LS mean ± SEM of six rats (percentage of relative abundance). Only classified genera with relative abundance above 5.0% cut-off level are shown. Groups were assessed by one-way ANOVA followed by Tukey's test. Means with different superscripts (lowercase letters) differ significantly (p <0.05)*.

Moreover, the relative abundance of *Desulfovibrio* was significantly higher in the starch+olive (negative control) group than other diet groups with FOS added. A significantly higher relative abundance of *Klebsiella* and *Ruminococcaceae_UCG-005* were observed in the galactose+FOS group and galactose, respectively, compared to the other diet groups. At 6 weeks, no similar difference was observed in *Klebsiella* and *Ruminococcaceae_UCG-005* (data not shown).

*Alloprevotella* abundance was significantly higher in the fructose+FOS and galactose groups than in the other diet groups. The relative abundance of unidentified_*Ruminococcaceae* was significantly higher in the galactose group than in the other diet groups except for starch (control) and fructose groups. Moreover, *Parasutterella* abundance was significantly higher in the starch+FOS compared to other diet groups. However, there were no differences in *Alloprevotella*, unidentified_*Ruminococcaceae*, and *Parasutterella* abundance at 6 weeks of intervention (data not shown).

**Figure 4:**
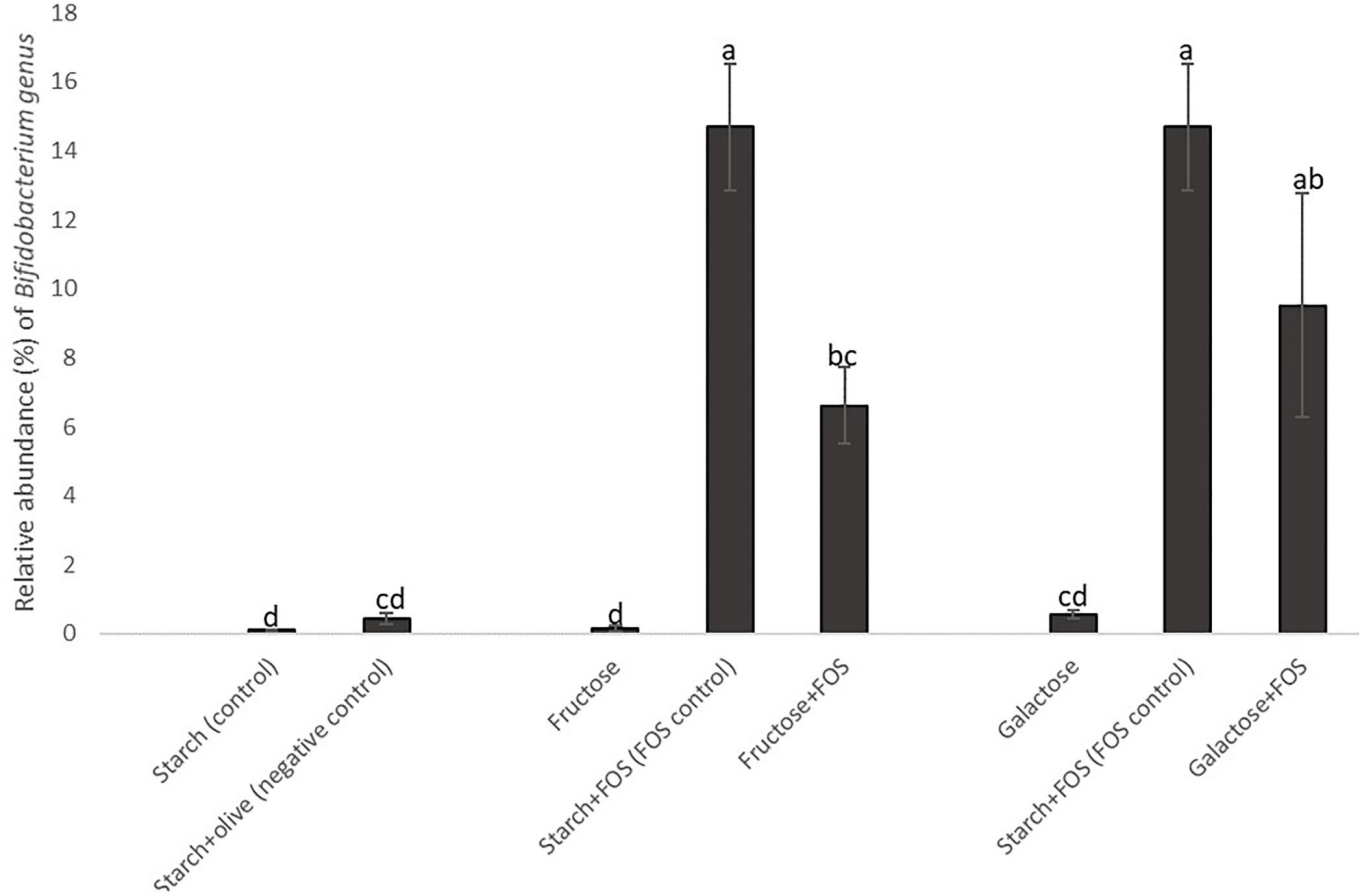
Changes in the relative abundance of the genus *Bifidobacterium* in the large intestine of rats fed a control diet (starch, starch+FOS, starch+olive) or a high-fructose or high-galactose diet, with and without added fructooligosaccharides (FOS), after 12 weeks. Values shown are LS mean ± SEM of six rats (percentage of relative abundance). Groups were assessed by one-way ANOVA followed by Tukey's test. Means with different superscripts (lowercase letters) differ significantly (*p* < 0.05).

In general, the taxon-based analysis showed marked changes in gut microbiota composition in diets with added FOS, but no obvious changes in groups fed a high-fructose or high-galactose diet without added FOS. The relative abundance of Actinobacteria was higher in all diet groups with added FOS, indicating that FOS significantly stimulated the growth of Actinobacteria. The genus *Bifidobacterium* was present in higher abundance in diet groups with added FOS than in diet groups without FOS (Figure 4), which contributed most of the increase of Actinobacteria at the phylum level. The relative abundance of *Bifidobacterium* was significantly higher in the starch+FOS and galactose+FOS groups than in all other diet groups except the fructose+FOS group after 12 weeks. At 6 weeks, a similar pattern of increased abundance of *Bifidobacterium* (*p* < 0.05) in rats fed diets with added FOS was observed (data not shown). In summary, the results showed that including FOS in the diet had an important effect on microbiota composition.

### Association between gut microbiota composition, metabolic factors, and inflammation and gut permeability markers after 12 weeks

In general, modest correlations were observed between the relative abundance of microbial phyla and selected metabolic factors and inflammation and gut permeability markers (Table 4). We found positive correlations between Firmicutes and Cyanobacteria and body weight, whereas Bacteroidetes and Actinobacteria were inversely associated with body weight. The relative abundance of Actinobacteria was inversely associated with endotoxin concentration but positively associated with lysine concentration. Verrucomicrobia composition was inversely associated with *N*ε-carboxy-methyl-lysine (CML) and pentosidine concentrations. Cyanobacteria composition was positively associated with endotoxin concentration after 12 weeks of intervention (Table 4).

**Table 4:** Spearman's rank correlation coefficient between gut microbiota composition (phylum level), metabolic factors, and inflammation and gut permeability markers after 12 weeks of intervention.

	**Firmicutes**	**Bacteroidetes**	**Actinobacteria**	**Verrucomicrobia**	**Proteobacteria**	**Tenericutes**	**Cyanobacteria**
Body weight (g)	0.541^***^	−0.373^*^	−0.446^**^	0.165	−0.130	0.076	0.505^***^
Metabolic factors							
Blood glucose (mg/dl)	0.025	0.060	0.033	0.170	−0.271	0.005	−0.071
Insulin (ng/dl) ^§^	0.100	−0.044	−0.028	−0.168	−0.004	−0.062	−0.009
HOMA-IR (mg/dl)	0.088	0.027	0.022	−0.078	−0.191	−0.047	−0.046
Inflammatory markers							
CRP (ng/ml)	0.093	0.186	−0.125	−0.275	0.123	0.199	0.184
IL-6 (pg/ml)	0.096	−0.240	0.052	0.188	−0.341	0.122	0.079
IL-1 β (pg/ml)	−0.046	−0.098	0.038	0.170	−0.139	−0.301	0.050
TNF-α (pg/ml)	−0.047	−0.044	0.200	0.107	−0.240	−0.024	−0.167
Advanced glycation end products (AGEs)-inflammation-related markers							
CML (ng/ml)	0.129	−0.078	0.077	−0.391^*^	−0.042	0.148	0.075
Pentosidine (ng/ml)	0.095	0.035	0.142	−0.381^*^	−0.104	0.089	−0.015
Lysine (ng/ml)	−0.264	−0.091	0.524^**^	−0.057	−0.015	0.118	−0.253
Gut permeability markers							
Endotoxin (pg/ml)	0.133	−0.148	−0.381^*^	0.202	0.247	0.164	0.432^**^
Zonulin (ng/ml)	−0.207	0.086	0.127	−0.024	−0.066	−0.177	−0.185

*CRP, c-reactive protein, IL-6, interleukin-6, IL-1β, interleukin-1β, TNF-α, tumor necrosis factor-α, CML, Nε-carboxy-methyl-lysine, HOMA-IR, Homeostatic Model Assessment, Insulin Resistance.*

* *p <0.05,*

** *p <0.01 and*

*** *p <0.001.*

§ *Sample analyzed in serum*.

Scatterplots for the phyla Firmicutes, Bacteroidetes, Actinobacteria, Verrucomicrobia, and Cyanobacteria with selected markers with significant correlations are presented in Supplementary Figure S1.

## Discussion

The results in the present study indicated that high-fructose and high-galactose diets did not have any consistent effect on microbiota composition at the phylum level under the conditions evaluated. Only groups treated with FOS showed consistent differences in microbiota composition, in particular, increased abundance of Actinobacteria which was mainly driven by an increase in the relative abundance of *Bifidobacterium*. However, we found that the gut microbiota was associated with several metabolic factors and biomarkers when data from all diet groups were pooled. To our knowledge, this is the first study to examine the effect on microbiota composition of high fructose and galactose intake, with and without added FOS to alleviate the negative effect of these sugars.

As reported in our previous study (44), the groups fed galactose or galactose+FOS had lower body weight than the other diet groups. This was accompanied by clinical symptoms in rats in these groups, including polyuria and lens opacity after high intake of galactose. Their energy expenditure could have been altered. Several studies have reported similar symptoms after feeding rats with diets containing 50% galactose (52, 53).

Dietary components strongly influence the richness and diversity of gut microbiota (14). The high richness and diverse microbiota have been associated with health benefits such as protection against enteropathogens, and contribute to normal immune function (54–56). To date, there is no uniform definition of a healthy gut microbiota composition, mainly due to large inter-individual variability resulting from differences in, for example, dietary and cultural habits, lifestyle, environment, and antibiotic use (57, 58). On the other hand, many studies have reported skewed microbial composition in several types of diseases. In many cases, this is referred to as dysbiosis, which is commonly associated with a reduction in microbial diversity, a decrease in the abundance of certain families within the order *Clostridiales*, and an increased abundance of Proteobacteria (39, 59). Increased proportions of Proteobacteria, and in particular, members of the Enterobacteriaceae, have been linked to host susceptibility to infection (39). For instance, it has been shown that dysbiosis shifts the abundance of Proteobacteria, an effect associated with metabolic syndrome and increased risk of diseases such as inflammatory bowel disease and cancers (60). Moreover, several studies have reported that a diet containing FOS significantly modulates species richness and diversity of gut microbiota (34, 43, 61, 62) and that FOS supplementation enhances the growth of unique bacteria and increases diversity (34, 62). In contrast, in our study species richness was significantly lower in starch+FOS than in other diet groups with added FOS after 12 weeks. The effect of diet on gut microbiota diversity was also lower in the starch+FOS group, as indicated by the Shannon index values in Figure 1B.

Several studies have reported that high doses of simple sugars are not cleared by the small intestine, and therefore reach the large intestine and alter gut microbiota composition in a direction associated with metabolic disorders (19, 63, 64). It is difficult to judge whether this is causative or not. High glucose and high fructose intake have been shown to increase Proteobacteria and decrease Bacteroidetes after a 12-week intervention in mice (40, 63). Proteobacteria are Gram-negative bacteria, with lipopolysaccharides (LPS) as structural components of their cell walls, and can rapidly utilize simple sugars (65). Increased growth of Proteobacteria may contribute to increased LPS load, which can alter tight junction proteins and increase intestinal permeability and infiltration of LPS into the bloodstream, inducing the release of cytokines and chemokines (63, 65, 66). Several studies in which D-galactose was used to induce aging in animal models have also reported an increased abundance of Bacteroidetes and lowered abundance of Firmicutes, Actinobacteria, Proteobacteria, and Cyanobacteria (27, 28, 67). However, our study did not show a clear effect of a high-galactose or high-fructose diet on the abundance of Bacteroidetes, Firmicutes, Actinobacteria, Verrucomicrobia, Proteobacteria, Tenericutes, and Cyanobacteria. Stimulating the expression of sodium/glucose transporter-1 (SGLT-1) by SCFAs may increase the absorption of monosaccharides in the small intestine (68). One possible explanation for the lack of effect in our study could be the action of SCFAs in enhancing the absorption of monosaccharides. However, we did not measure the production of SCFA and cannot confirm this suggestion.

Numerous studies have reported beneficial health effects of readily fermentable FOS. FOS intake is widely known to selectively modulate the composition of gut microbiota, especially Actinobacteria (62). Actinobacteria was one of seven major phyla found in rats in this study. Although the abundance of Actinobacteria in the gut is generally low, they play a very important role in human health, including maintenance of gut homeostasis (69). The most prevalent genus in this phylum is *Bifidobacterium*, which is widely used as a probiotic and has been inversely associated with various pathological conditions such as obesity and diabetes (69, 70). Similar results were observed in our study, where the rats fed diets containing FOS had a significantly higher abundance of Actinobacteria and *Bifidobacterium* (Figures 3, 4). *Bifidobacterium* has also been associated with the improvement of gut integrity by enhanced expression of tight junction proteins (66, 71). However, we found no significant association between *Bifidobacterium* and gut permeability markers (zonulin and endotoxin concentrations) (Supplementary Table S1).

In our study, the starch+FOS diet significantly increased Verrucomicrobia abundance (Figure 2). This phylum is primarily dominated by the genus *Akkermansia*, which has been studied for its role in the regulation of the immune system, intestinal integrity, peptide secretion, and inflammation (72). *Akkermansia muciniphila* is involved in the expression of IFNγ-regulated gene and glucose parameters in the gut, and improves glucose metabolism including glucose tolerance and fasting glucose in both animal and human models (73). A higher abundance of *A. muciniphila* has been linked with healthier metabolic status and improvement in glucose homeostasis and blood lipids (74). However, our results did not support these findings (Supplementary Table S1). In addition, a significantly increased abundance of *Ruminococcus gnavus* from Firmicutes phyla was also observed in the group fed the starch+FOS diet (Table 3, Figure 3). Diet has shaped the composition of *R. gnavus* through the metabolism of FOS and degradation of resistant starch in the gut (75, 76). Different combinations of starch and sugars affect the abundance of *Ruminococcus_gnavus* (76). In a cross-feeding study, non-digestible carbohydrates supplied have modulated inhabiting the mucus niche by *R. gnavus* (77). Moreover, several studies have demonstrated the roles of *R. gnavus* in various disease conditions such as multiple myeloma, myelodysplastic, and fecal peritonitis (78, 79).

Recent studies have investigated the association between gut microbiota and various clinical parameters (69, 80, 81). We analyzed the association between seven major phyla (Firmicutes, Bacteroidetes, Proteobacteria, Cyanobacteria, Actinobacteria, Verrucomicrobia, and Tenericutes) and selected metabolic parameters and inflammation and gut permeability markers. The major phylum Firmicutes showed a positive correlation with body weight in this study. Human and animal studies have consistently reported that a high abundance of Firmicutes over Bacteroidetes is associated with a decrease in body weight (81, 82). In contrast, our study demonstrated a positive correlation between Firmicutes and body weight. At the genus level, *Bacteroides* is beneficial for glucose metabolism through improvements in glucose tolerance and insulin resistance (83, 84). We observed an association between Actinobacteria and body weight, lysine, and endotoxin concentrations (Table 4). A recent study reported a protective effect of Verrucomicrobia against the development of metabolic diseases (72). Our study demonstrated an inverse association between Verrucomicrobia and CML and pentosidine concentrations. In phylogenic analyses, diets high in AGEs have been shown to reduce Verrucomicrobia abundance (85). Cyanobacteria has been reported to have toxicity and pathological effects on human health, including gastrointestinal health and respiratory diseases (86). Various toxics such as endotoxin, hepatotoxin, and neurotoxins produced by Cyanobacteria may affect body organs (86, 87). Our study demonstrated a positive association between Cyanobacteria and body weight and an inverse association between Cyanobacteria abundance and lysine concentration.

The present study had some limitations. First, free fructose and free galactose were used although most sugars are consumed as sucrose and lactose. Second, the use of a high dose of fructose and galactose may have generated a mild toxic effect on the rats. Third, interpretation of the results is more challenging due to modification of the study design as the rats fed the galactose and galactose+FOS demonstrated clinical symptoms and the intervention had to be adapted. Fourth, the present results in a rat model cannot be directly translated into humans. Fifth, the SCFAs profile was not analyzed which could have linked FOS-related gut microbiota composition and activity with metabolic outcomes. However, the present investigation did not include the data on metabolic changes that might relate to the microbiota communities measured. The reason for such exclusion was that there were no substantial effects on metabolic parameters in response to the interventions. Nevertheless, our study also has several strengths. First, the study was large and the effects of different sugars with and without FOS under isocaloric conditions were compared in the same study. Second, by using a rat model, we could evaluate direct intestinal samples to understand the modulation of gut microbiota which is rare in studies of the gut microbiota where fecal samples are typically used and fecal samples do not normally correlate with intestinal samples. Third, in this study, we were able to establish cause and effects between fructose, galactose, FOS, and the gut microbiota and not merely associations as in many other studies.

In summary, we did not find any coherent effect of high intake of galactose or fructose compared with all other diets on gut microbiota composition after 12 weeks. Thus, the data did not support our hypothesis of the direct effects of galactose or fructose on gut microbiota, mediating adverse metabolic effects of these sugars. However, adding FOS to the sugar diets increased the abundance of the genus *Bifidobacterium* in the phylum Actinobacteria in the rats, supporting our second hypothesis. On pooling the data from the different diet groups, we found modest correlations between major phyla in the gut microbiota and several metabolic factors and inflammation-related markers, confirming the reported link between gut microbiota and cardiometabolic risk factors. Further studies should investigate the impact of gut microbiota activity as measured by different metabolites such as SCFAs, and their relation to cardiometabolic risk factors.

## Data availability statement

The datasets presented in this study can be found in online repositories. The names of the repository/repositories and accession number(s) can be found below: https://www.ncbi.nlm.nih.gov/bioproject/PRJNA838460.

## Ethics statement

The animal study was reviewed and approved by Regional Council Stuttgart (Baden-Württemberg, Germany) Ethics Committee.

## Author contributions

NM designed and conducted the experiments, performed sampling, statistical analysis, and interpretation of results, and wrote the manuscript. JD conducted microbiota analysis, interpreted results, and participated in manuscript writing. JK conducted the experiments. GZ, KM, and AW contributed to designing the experiments and critically reviewed the manuscript. JF was involved in designing the study, conducting the rat trial, interpretation of the results, and manuscript writing. RL formulated the hypotheses and designed the study, contributed to the interpretation of results and manuscript writing, and had overall responsibility for the study. All authors contributed to the article and approved the submitted version.

## Funding

This project has received funding from FORMAS under the umbrella of the European Joint Programming Initiative A Healthy Diet for a Healthy Life (JPI HDHL) and of the ERA-NET Cofund HDHL INTIMIC (GA No. 727565 of the EU Horizon 2020 Research and Innovation Programme), Swedish Research Council (Dnr 2017-05840; Formas Dnr 2016-003114), and by a starting grant from Chalmers Foundation. NM was funded by a scholarship from the Ministry of Higher Education Malaysia and Universiti Malaysia Pahang.

## Conflict of interest

The authors declare that the research was conducted in the absence of any commercial or financial relationships that could be construed as a potential conflict of interest.

## Publisher's note

All claims expressed in this article are solely those of the authors and do not necessarily represent those of their affiliated organizations, or those of the publisher, the editors and the reviewers. Any product that may be evaluated in this article, or claim that may be made by its manufacturer, is not guaranteed or endorsed by the publisher.
