# Supplementary material for: Effect of a diet rich in galactose or fructose, with or without fructooligosaccharides, on gut microbiota composition in rats
Source: Front Nutr. 2022 Aug 12;9:922336. doi: 10.3389/fnut.2022.922336 (PMC9412906; doi:10.3389/fnut.2022.922336)
Supplement: Supplementary file 1 [file Table_1.DOCX]

| **Table S1.** Spearman's rank correlation coefficient between gut microbiota composition (genus level) with metabolic factors, inflammatory- and gut permeability- markers | | | | | | | | | |
| --- | --- | --- | --- | --- | --- | --- | --- | --- | --- |
|  |  |  |  | |  |  |  |  |  |
|  | *Clostridium_ sensu_stricto_1* | *Lachnospiraceae_NK4A136_group* | *Romboutsia* | | *Bifidobacterium* | *Akkermansia* | *[Ruminococcus] _gnavus_group* | *Turicibacter* | *Desulfovibrio* |
|  |  |  |  | |  |  |  |  |  |
| Body weight (g) | -0.175 | 0.387* | 0.347* | | -0.450** | 0.138 | -0.352* | 0.057 | 0.197 |
|  |  |  |  | |  |  |  |  |  |
| *Metabolic factors* |  |  |  | |  |  |  |  |  |
| Blood glucose (mg/dL) | -0.060 | -0.146 | 0.135 | | 0.021 | 0.143 | 0.049 | 0.166 | -0.152 |
| Insulin (ng/dL)^§^ | -0.193 | 0.118 | 0.078 | | -0.025 | -0.208 | -0.089 | 0.444** | -0.084 |
| HOMA-IR (mg/dL) | -0.158 | -0.007 | 0.116 | | 0.021 | -0.123 | -0.065 | 0.480** | -0.145 |
|  |  |  |  | |  |  |  |  |  |
| *Inflammatory markers* | |  |  |  | |  |  |  |  |
| CRP (ng/mL) | 0.159 | 0.261 | -0.023 | | -0.125 | -0.328 | -0.138 | 0.129 | 0.298 |
| IL-6 (pg/mL) | 0.006 | 0.040 | -0.099 | | 0.031 | 0.275 | 0.011 | -0.022 | -0.142 |
| IL-1β (pg/mL) | -0.108 | 0.027 | 0.026 | | 0.046 | 0.189 | -0.156 | -0.168 | -0.164 |
| TNF-α (pg/mL) | -0.122 | -0.226 | -0.116 | | 0.192 | 0.150 | 0.243 | 0.055 | -0.360* |
|  |  |  |  | |  |  |  |  |  |
| *Advanced glycation end products (AGEs)-inflammation-related markers* | | | |  | |  |  |  |  |
| CML (ng/mL) | 0.158 | 0.138 | -0.321 | | 0.036 | -0.361* | 0.017 | -0.002 | 0.070 |
| Pentosidine (ng/mL) | 0.188 | 0.028 | -0.398* | | 0.103 | -0.367* | 0.101 | -0.009 | -0.032 |
| Lysine (ng/mL) | -0.115 | -0.106 | -0.106 | | 0.481** | -0.057 | 0.278 | -0.266 | -0.255 |
|  |  |  |  | |  |  |  |  |  |
| *Gut permeability markers* | |  |  |  | |  |  |  |  |
| Endotoxin (pg/mL) | -0.352* | 0.250 | 0.490** | | -0.385* | 0.202 | -0.328* | -0.258 | 0.289 |
| Zonulin (ng/mL) | -0.162 | 0.116 | -0.205 | | 0.156 | -0.024 | -0.005 | 0.093 | -0.029 |
|  |  |  |  | |  |  |  |  |  |
|  | *Alistipes* | *Klebsiella* | *Bacteroides* | | *Ruminococcaceae_UCG-005* | *Alloprevotella* | *Blautia* | *unidentified_ Ruminococcaceae* | *Parasutterella* |
|  |  |  |  | |  |  |  |  |  |
| Body weight (g) | -0.162 | -0.410** | -0.472** | | 0.126 | -0.436** | -0.309 | -0.068 | -0.423** |
|  |  |  |  | |  |  |  |  |  |
| *Metabolic factors* |  |  |  | |  |  |  |  |  |
| Blood glucose (mg/dL) | 0.034 | 0.006 | -0.000 | | -0.109 | -0.035 | -0.102 | -0.137 | 0.042 |
| Insulin (ng/dL)^§^ | 0.081 | 0.483** | -0.022 | | -0.034 | 0.253 | 0.127 | 0.155 | -0.065 |
| HOMA-IR (mg/dL) | 0.100 | 0.431** | -0.003 | | -0.076 | 0.194 | -0.005 | 0.013 | -0.044 |
|  |  |  |  | |  |  |  |  |  |
| *Inflammatory markers* | |  |  |  | |  |  |  |  |
| CRP (ng/mL) | 0.292 | -0.017 | 0.031 | | 0.138 | 0.146 | -0.108 | 0.232 | -0.153 |
| IL-6 (pg/mL) | -0.120 | -0.020 | 0.013 | | 0.104 | -0.046 | 0.095 | -0.041 | -0.064 |
| IL-1β (pg/mL) | -0.141 | -0.077 | -0.188 | | -0.165 | -0.184 | -0.318 | -0.324 | -0.065 |
| TNF-α (pg/mL) | -0.193 | 0.169 | 0.195 | | -0.110 | 0.143 | 0.254 | -0.061 | 0.225 |
|  |  |  |  | |  |  |  |  |  |
| *Advanced glycation end products (AGEs)-inflammation-related markers* | | | |  | |  |  |  |  |
| CML (ng/mL) | 0.068 | 0.148 | 0.117 | | 0.279 | -0.016 | 0.089 | 0.091 | -0.078 |
| Pentosidine (ng/mL) | 0.177 | 0.212 | 0.231 | | 0.242 | 0.097 | 0.151 | 0.133 | 0.035 |
| Lysine (ng/mL) | -0.143 | 0.248 | 0.194 | | -0.331* | -0.138 | -0.179 | -0.351* | 0.338* |
|  |  |  |  | |  |  |  |  |  |
| *Gut permeability markers* | |  |  |  | |  |  |  |  |
| Endotoxin (pg/mL) | -0.064 | -0.174 | -0.258 | | 0.085 | -0.262 | -0.361* | -0.084 | -0.284 |
| Zonulin (ng/mL) | 0.114 | 0.154 | 0.042 | | -0.093 | 0.175 | -0.101 | 0.138 | -0.009 |
|  |  |  |  | |  |  |  |  |  |
| CRP - c-reactive protein, IL-6 - interleukin-6, IL-1β - interleukin-1β, TNF-α - tumor necrosis factor-α, CML - *N*ε-carboxy-methyl-lysine, HOMA-IR - Homeostatic Model Assessment -Insulin Resistance. (**P*<0.05, ***P*<0.01 and ****P*<0.001). § sample analyzed in serum. | | | | | | | | | |


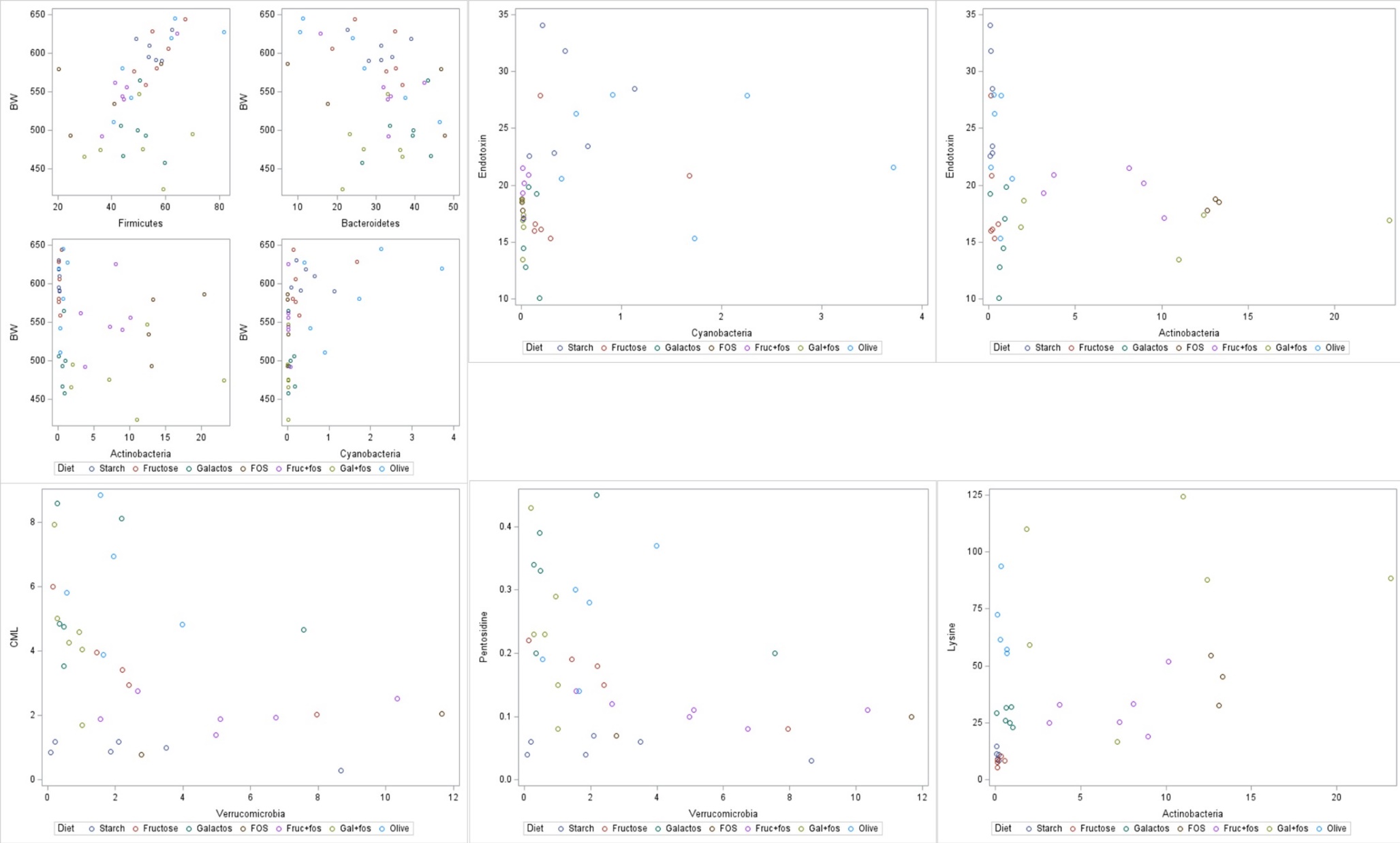


**Figure S1**. Scatterplot of the abundance of the phyla Firmicutes, Bacteroidetes Actinobacteria, Verrucomicrobia, and Cyanobacteria, plotted against metabolic factors and gut permeability markers, in rats with high fructose or galactose intake, with and without added fructooligosaccharides (FOS), after 12 wk
